# Detection of *Felis catus* Gammaherpesvirus 1 in Domestic Cat Saliva: Prevalence, Risk Factors, and Attempted Virus Isolation

**DOI:** 10.3390/pathogens13020111

**Published:** 2024-01-26

**Authors:** Malcolm A. M. Hill, Tracy Satchell, Ryan M. Troyer

**Affiliations:** 1 Department of Microbiology and Immunology, University of Western Ontario, London, ON N6A 5C1, Canada; mhill93@uwo.ca; 2 London Animal Care Centre, London, ON N6K 3T6, Canada

**Keywords:** virus, herpesvirus, propagation, felid, feline, feral, *Percavirus*, transmission, saliva

## Abstract

*Felis catus* gammaherpesvirus 1 (FcaGHV1) infects domestic cats worldwide, yet it has not been successfully propagated in cell culture, and little is known about how it is shed and transmitted. To investigate the salivary shedding of FcaGHV1, we quantified FcaGHV1 DNA in feline saliva by qPCR. For FcaGHV1-positive saliva, we sequenced a portion of the viral glycoprotein B (gB) gene and attempted to isolate the infectious virus by passage in several felid and non-felid cell lines. We detected FcaGHV1 DNA in 45/227 (19.8%) saliva samples with variable viral DNA loads from less than 100 to greater than 3 million copies/mL (median 4884 copies/mL). Multiple saliva samples collected from an infected cat over a two-month period were consistently positive, indicating that chronic shedding can occur for at least two months. Cat age, sex, and health status were not associated with shedding prevalence or viral DNA load in saliva. Feral status was also not associated with shedding prevalence. However, feral cats had significantly higher FcaGHV1 DNA load than non-feral cats. Sequencing of FcaGHV1 gB showed low sequence diversity and >99.5% nucleotide identity to the worldwide consensus FcaGHV1 gB sequence. We did not detect virus replication during the passage of FcaGHV1-positive saliva in cell culture, as indicated by consistently negative qPCR on cell lysate and supernatant. To our knowledge, these data show for the first time that cats in Canada are infected with FcaGHV1. The data further suggest that shedding of FcaGHV1 in saliva is common, can occur chronically over an extended period of time, and may occur at higher levels in feral compared to non-feral cats.

## 1. Introduction

The *Herpesviridae* family of large DNA viruses includes three subfamilies: *Alpha*-, *Beta*-, and *Gammaherpesvirinae*. Among these, the gammaherpesviruses (GHVs) are lymphotropic and responsible for infection of diverse vertebrate and invertebrate host species [1]. Although GHVs are known to infect a wide range of species, individual GHVs typically have a relatively host-specific tropism [1,2]. A hallmark of GHVs is their predisposition to enter into a latent state upon infection of the host cell, allowing the virus to evade host immunity and persist for years [3]. The latent phase of GHVs must be closely regulated to ensure a proper balance with the virus’s lytic phase. The lytic state of GHVs is necessary for transmission from host to host but leaves the virus vulnerable to detection and clearance by the host immune system [3]. As a result, GHVs have evolved to remain largely latent, with episodic reactivation of lytic replication resulting in virus shedding and transmission. Few GHVs are pathogenic under natural conditions in their adapted hosts. However, under conditions of immunosuppression or transfer to a non-adapted host, GHVs can cause a variety of severe disease conditions in humans and animals, often involving lymphocyte proliferation [4,5,6,7,8].

*Felis catus* gammaherpesvirus 1 (FcaGHV1), classified taxonomically as felid gammaherpesvirus 1, is a member of the *Percavirus* genus with a close phylogenetic relationship to other recently identified GHVs of carnivores [9]. FcaGHV1 was discovered in 2014 through PCR screening for the GHV DNA polymerase gene and glycoprotein B gene in blood samples from domestic cats, with a prevalence of approximately 16% among cats in the United States [10]. Further studies detected viral DNA in domestic cats in Australia, Europe, Asia, and South America, with a prevalence between 1 and 24% [11,12,13,14,15,16,17,18]. However, serologic detection of antibodies to FcaGHV1 suggests that PCR on blood may underestimate the prevalence of infection [19]. With regard to host tissue and cellular tropism, FcaGHV1 has been found to infect a range of tissues [18] and, in blood, infects both B and T cells, a relatively unusual characteristic as GHVs typically have specific tropism for one or the other [20]. Notably, FcaGHV1 DNA and/or viral transcripts have been identified in tumor tissue from several cats suffering from neoplasia [21,22,23,24], but thus far, no clear link between FcaGHV1 and specific cancers has been established [25]. Studies have identified several risk factors for FcaGHV1 DNAemia in blood, including most notably male sex, older age, poor health condition, infection with feline immunodeficiency virus (FIV), and infection with hemotropic mycoplasma [10,12,13,17,18]. While positive associations with male sex and FIV infection suggest that fighting and biting behaviors may play a role in FcaGHV1 cat-to-cat transmission, modes of shedding and transmission remain unknown.

Herpesviruses can be shed in a variety of body fluids and may be transmitted via oral, nasal, ocular, genital, and respiratory routes, as well as through the transfer of blood and tissues. Of these routes, shedding and transmission through saliva is highly common. All eight human herpes viruses are shed in saliva [26,27,28,29,30]. Likewise, many animal herpesviruses can be shed and transmitted via saliva [31,32,33,34], including feline herpesvirus type-1, an alphaherpesvirus of cats [35]. Recently, Rose et al. [22] detected FcaGHV1 DNA in oropharyngeal swabs and salivary gland epithelial tissue of cats, suggesting salivary shedding of the virus as a potential mechanism of transmission. While these findings provide strong evidence for salivary shedding, the prevalence and risk factors of FcaGHV1 shedding in feline saliva have not been well characterized. Furthermore, FcaGHV1 has not been propagated in cell culture, severely limiting our ability to study viral infection. To address these knowledge gaps, we tested saliva from domestic cats for FcaGHV1 DNA to characterize the prevalence and risk factors associated with FcaGHV1 shedding. We also attempted to propagate FcaGHV1 from viral DNA-positive feline saliva in a range of cell lines.

## 2. Materials and Methods

### 2.1. Saliva Samples

Saliva samples were collected and donated by veterinarians at the municipal Companion Animal Hospital, London Animal Shelter Services, as part of the regular evaluation of stray cats under anesthesia for sterilization or medical care. Saliva samples from privately owned cats were donated by the owner. Saliva was collected with sterile cotton swabs on the inside of the cheeks and sub-lingual region, and swabs were stored at −20 °C prior to donation. Each cotton tip was placed tip-side-down in a trimmed P1000 pipette tip inside a 1.5 mL microtube such that saliva, but not the swab, could drain through the tapered end of the pipette tip into the microtube upon centrifugation at 1000× *g* for 2 min. Sample volumes were recorded, sealed tightly with parafilm, and stored at −20 °C until further processing. Cat feral status was determined by cat behavior in the shelter as an indicator of their degree of socialization with humans using the structured assessments of Slater et al. [36].

### 2.2. Nucleotide Purification

Nucleotide purification was performed using Qiagen DNeasy Blood and Tissue kit reagents (Valencia, CA, USA). For cat saliva, each sample was thawed and volume-corrected to 180 µL with sterile PBS. For cell lysate/supernatant from the virus propagation experiment, each sample was thawed, and 180 µL was used for extraction. DNA was extracted from each sample according to the kit manufacturer’s instructions prior to the DNA elution step. Purified nucleotides were eluted with 50 µL buffer AE warmed to 56 °C and centrifuged at 6000× *g* for 1 min. The elution process was repeated with an additional 50 µL buffer AE to yield a total volume of 100 µL. Ethanol precipitation was next conducted by adding 2 µL glycogen, 10 µL 3M sodium acetate, and 250 µL 100% ethanol pre-chilled to −80 °C. The sample was stored at −80 °C for two hours before centrifugation at 4 °C at 14,000 rpm for 20 min. The supernatant was discarded, and the pellet was allowed to air-dry for 15 min. The sample was resuspended in 20 µL sterile ddH_2_O, and DNA was quantified using a NanoDrop 1000 UV-Vis spectrophotometer (NanoDrop Technologies, Wilmington, DE, USA).

### 2.3. Real-Time Quantitative PCR (qPCR)

FcaGHV1 DNA was detected and quantified using a previously described qPCR targeted to the gB gene of FcaGHV1 [10]. Briefly, 25 µL qPCR reaction mixtures were prepared on ice with TaqMan Fast Advanced master mix (Applied Biosystems, Waltham, MA, USA), 400 nM primers FGHV-F3 (ACATCTTCACTGGACAACTGG), and FGHV-R3 (GTGCATTTGATGTCCTGACTG), 200 nM probe FGHV-P3 (TGAACAGCTGAGTCTCTACAAGTCTCCA) labelled with 6-FAM reporter dye and ZEN-Iowa Black FQ quenchers (IDT, Coralville, IA, USA), and 5 µL purified DNA. Samples were loaded in duplicate into a 0.1 mL 96 well qPCR plate and run under the following conditions in a QuantStudio 5 real-time PCR system (Applied Biosystems): 5 min at 95 °C, followed by 45 cycles of 5 s at 95 °C and 1 min at 60 °C. Both ddH_2_O and purified human saliva were used as negative controls. Plasmid standards for quantification were prepared as previously described by Troyer et al., and FcaGHV1 DNA load was calculated as DNA copies/mL saliva [10]. Amplification efficiency for all assays was consistently 90 to 110%. Samples were considered positive if reactions had a cycle threshold (Ct) of ≤35.0 and a quantity of more than 3 copies per reaction.

### 2.4. PCR Amplification, DNA Sequencing, and Alignment of FcaGHV1 gB

The FcaGHV1 gB gene was amplified from saliva DNA by nested PCR. First-round PCR reaction mixtures of 50 µL contained Taq FroggaMix (FroggaBio, Toronto, ON, Canada), 400 nM primers FGHV-F1 (ACCTGCACCAGAGCATGAGA), and FGHV-R1 (TGTCCAGTACGTTAGCCAATCTTT), and 5 µL saliva DNA. Reaction cycling was performed using a SimpliAmp Thermocycler (ThermoFisher, Carlsbad, CA, USA) and included a denaturation step of 94 °C for 2 min, followed by 35 cycles of 94 °C for 30 s, 57 °C for 30 s, and 72 °C for 30 s; followed by an extension step at 72 °C for 5 min. Second-round PCR reaction mixtures were prepared with Taq FroggaMix (FroggaBio) and 400 nM primers FGHV-F2 (TACTCCAGACCCATCGTCACAT) and FGHV-R2 (TCGACTACCTCAAAGTCAATGTTTTC). Under sterile conditions, 2 µL of the first-round PCR product was added to 48 µL of the second-round reaction mix and amplified using cycling conditions identical to the first-round PCR. Second-round PCR products were purified using the QIAquick PCR purification kit (Qiagen) and Sanger sequenced by The Centre for Applied Genomics at The Hospital for Sick Children (Toronto, ON, Canada). After removal of the primer sequence, 224 nt unique gB sequences were aligned to reference sequences using the MUSCLE algorithm as implemented in the SnapGene v6.0.2 software package (GSL Biotech, San Diego, CA, USA) to produce a consensus FcaGHV1 gB sequence and identify nucleotide polymorphisms [37]. The 21 unique gB sequences from this study were deposited in GenBank https://www.ncbi.nlm.nih.gov/genbank/ (accessed on 15 January 2024) under accession numbers PP135521-PP135541.

### 2.5. Cell Culture

Cells obtained from the American Type Culture Collection (ATCC) included Crandell-Rees feline kidney (CRFK) cells (CCL-94), the feline embryonal tongue cell line Fc3Tg (CCL-176), African green monkey Vero cells (CCL-81), and mink lung epithelial cell line Mv1Lu (CCL-64). Newborn primary feline kidney (NPFK) cells from a domestic cat were kindly provided by Dr. Sue VandeWoude, Colorado State University. Minimally-passaged stocks of each cell line were maintained in liquid nitrogen, and all experiments were initiated using low-passage stocks. All cells were cultured in “complete” DMEM containing 10% Fetal Bovine Serum, 4.5 g/L Glucose with L-Glutamine and sodium pyruvate (Wisent, Saint-Jean-Baptiste, QC, Canada), supplemented with 10% Fetal Bovine Serum and 1X Penicillin-Streptomycin, with incubation at 37 °C with 5% CO_2_.

### 2.6. FcaGHV1 Isolation in Cell Culture

Attempts to isolate FcaGHV1 from saliva were initiated by culturing FcaGHV1 DNA-positive saliva on CRFK, Fc3Tg, NPFK, Vero, and Mv1Lu cells. Briefly, 50 µL of saliva was diluted in 950 µL warm complete DMEM (10% FBS, 1X penicillin-streptomycin, with 4.5 g/L L-Glutamine and sodium pyruvate, and 2.5 µg/mL Amphotericin B anti-fungal) and added to each cell monolayer in 12-well plates at 80% confluency. Cells were incubated at 37 °C in 5% CO_2_ alongside negative control wells inoculated with 50 µL FcaGHV1 DNA-negative saliva. Cells were monitored by light microscopy for CPE every 2–3 days. At 7 days post-inoculation, cells and media were collected by cell scraping, cells were lysed by two freeze-thaw cycles at −80 °C, cell lysate was centrifuged at 500× *g* for 10 min to pellet cellular debris, and the supernatant was collected. To initiate the next cell passage, 250 µL of supernatant was diluted in 750 µL of warm complete DMEM and added to each cell monolayer in 12-well plates at 80% confluency, and cells were incubated and monitored for 7 days alongside negative control wells as described for the initial passage. The remaining supernatant was stored at −80 °C for DNA extraction and qPCR testing. Cell passage was continued using identical procedures for up to 12 additional passages. In a separate propagation attempt, FcaGHV1 DNA-positive saliva was passaged on the same cell lines in the presence of the phorbol ester 12-O-tetradecanoylphorbol-13-acetate (TPA) at 200 µm (Cell Signaling Technology, Danvers, MA, USA).

### 2.7. Statistical Analysis

Statistical analysis was conducted with GraphPad Prism 9.0 (San Diego, CA, USA). Chi-squared tests were used to evaluate the association between FcaGHV1 DNA positivity and feline demographics. FcaGHV1 DNA copies/mL data was assessed by the Shapiro-Wilk test and found to be non-normally distributed. Nonparametric two-tailed Mann-Whitney tests were conducted to compare the distribution of FcaGHV1 DNA copies/mL saliva between feline demographic groups.

## 3. Results

### 3.1. Prevalence of FcaGHV1 DNA in Domestic Cat Saliva and Association with Potential Predictor Variables

**Figure 1:**
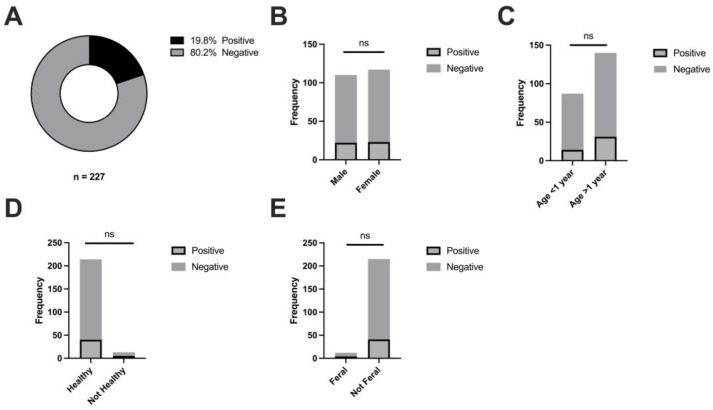
Prevalence of FcaGHV1 DNA in domestic cat saliva and association with potential predictor variables. (**A**) qPCR screening of 227 domestic cat saliva samples for FGHV DNA showed an overall positivity rate of 45/227 or 19.8%. Potential association between saliva GHV DNA status and potential predictor variables, including sex (**B**), age (**C**), feral status (**D**), and health status (**E**). Chi-square tests indicated no significant relationships between saliva GHV DNA status and the variables (ns).

**Figure 2:**
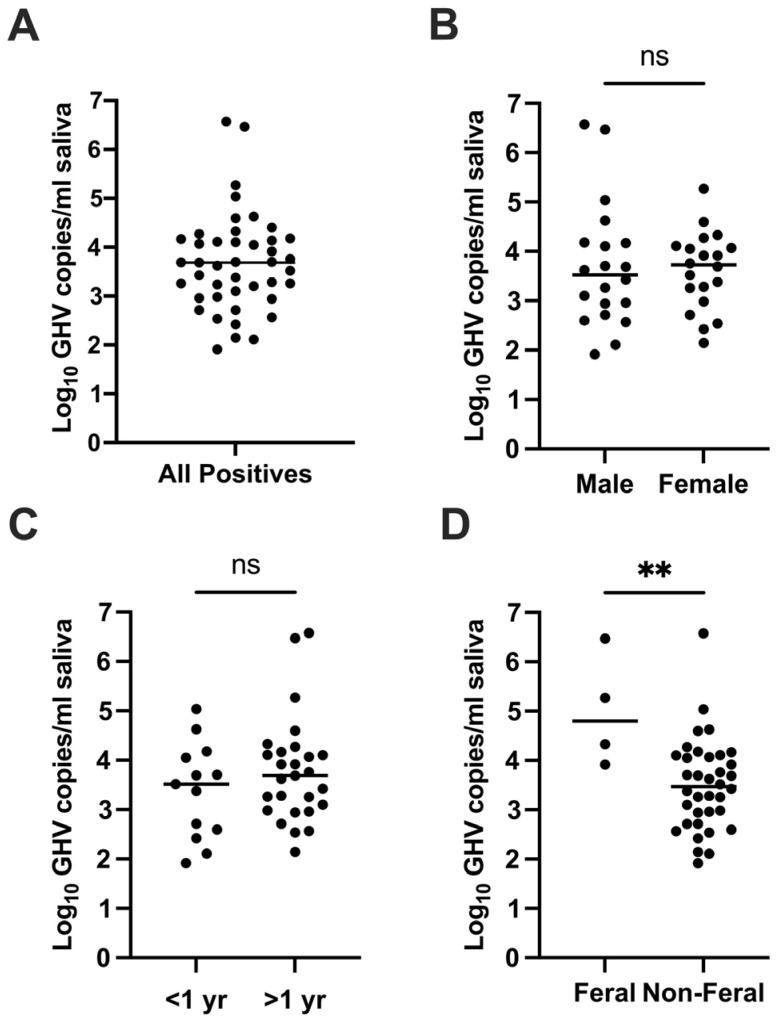
FcaGHV1 DNA loads and potential shedding risk factors in domestic cat saliva. (**A**) GHV DNA load in all positive saliva ranged between 82 and 3,755,742 copies/mL with a median of 4884 copies/mL. Data was assessed by the Shapiro-Wilk test and found to be non-normally distributed. Nonparametric two-tailed Mann-Whitney tests indicated no significant difference between the GHV DNA loads of (**B**) male versus female cats and (**C**) cats under one year of age versus cats over one year of age. (**D**) GHV DNA load was higher in feral cats compared to non-feral cats. ** *p* < 0.01; ns, not significant.

To investigate FcaGHV1 salivary shedding by domestic cats, we obtained saliva samples from 227 cats located in London, Ontario, Canada, and surrounding areas. The samples included 221 from cats at an animal shelter and 6 from private cat owners. We isolated DNA from saliva samples and quantified FcaGHV1 genomic DNA using a sensitive and specific qPCR targeted to the glycoprotein B (gB) gene of FcaGHV1 [10] and used extensively in previous studies [12,13,16,17,18,20,24,38,39]. We detected FcaGHV1 DNA in 45/227 samples, or 19.8% (Figure 1A). To our knowledge, this represents the first detection of FcaGHV1 in Canada. Stratification of results by sex, age, feral status, and health status is detailed in Table 1. The average age of all cats was found to be 2.05 years (range: 3 months to 198 months), with similar distributions between male and female cats. Cats with FcaGHV1-positive saliva averaged 2.48 years, and cats with FcaGHV1-negative saliva averaged 1.95 years. Adverse reported health conditions included upper respiratory infection, tumors, or heart conditions. Analysis for a relationship between these demographic factors and FcaGHV1 DNA saliva positivity showed no significant relationships (Figure 1B–E). Calculation of FcaGHV1 DNA load in saliva indicated a wide range of levels between 82 and 3,755,742 copies/mL with a median of 4884 copies/mL (Figure 2A). There were no significant differences between the DNA loads of positive samples stratified by sex or age (Figure 2B,C). In contrast, we found that feral cats had significantly higher FcaGHV1 DNA loads in saliva than non-feral cats (Figure 2D).

**Figure 3:**
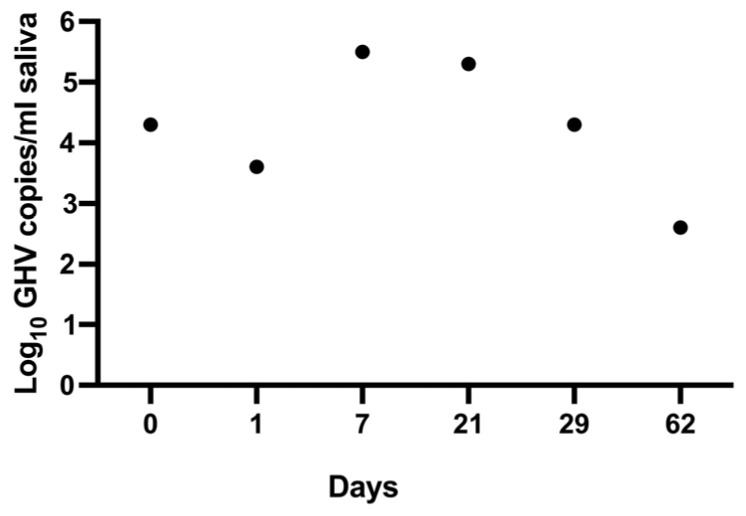
Chronic salivary shedding of FcaGHV1 DNA in a 12-year-old female domestic cat. qPCR screening of saliva found FcaGHV1 DNA consistently over two months with varying viral load (mean of 4.24 ± 1.10 log_10_ DNA copies/mL of saliva).

While these data indicate the occurrence of salivary FcaGHV1 shedding at a single point in time for many cats, we were also interested in investigating whether cats can chronically shed FcaGHV1 DNA over time. We had the opportunity to test saliva from a privately owned domestic 12-year-old female cat six times over a period of two months and found consistent salivary shedding of FcaGHV1 DNA between a range of 398 and 316,228 copies/mL with a mean value of 17,458 ± 12.5 copies/mL (4.24 ± 1.098 log_10_; Figure 3). Collectively, these results indicate that FcaGHV1 is present in Canadian domestic cats, salivary shedding of FcaGHV1 is common in cats, feral cats may have higher levels of viral shedding than non-feral cats, and a domestic cat can chronically shed FcaGHV1.

### 3.2. Sequence Analysis of FcaGHV1 gB from Saliva

**Figure 4:**
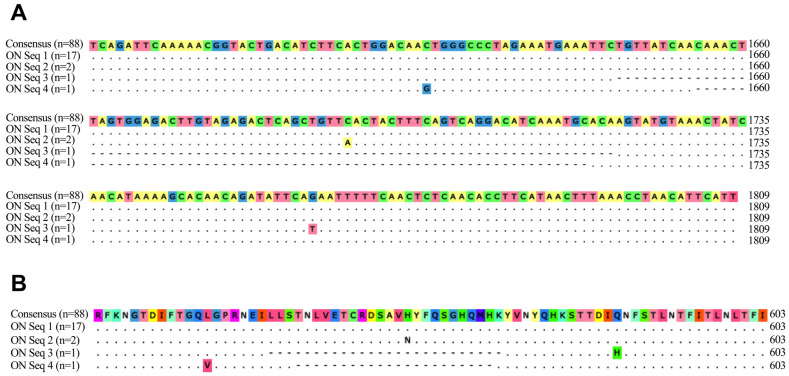
FcaGHV1 gB nucleotide (**A**) and amino acid (**B**) alignments. The 21 gB sequences from Ontario cat saliva (labeled ON) included four sequence variants (labeled Seq 1, Seq 2, etc.). Sequences are aligned to a consensus sequence generated from 88 FcaGHV1 gB sequences available in the NCBI sequence database. Alignments were conducted using the MUSCLE algorithm as implemented in the SnapGene v6.0.2 software package (GSL Biotech, San Diego, CA, USA).

As this study represents the first detection of FcaGHV1 in Canada to our knowledge, we sought to investigate the genetic diversity of FcaGHV1 in southwestern Ontario cats and compare these sequences to FcaGHV1 sequences from around the world. For this purpose, we selected 21 FcaGHV1-positive saliva samples for PCR amplification and sequencing of a 224 nt portion of the gB gene. We successfully amplified and sequenced gB for all 21 samples and aligned these sequences to 88 published FcaGHV1 gB sequences from the NCBI database (www.ncbi.nlm.nih.gov, accessed on 18 December 2023). Genetic diversity within this region of gB was low for all worldwide FcaGHV1 isolates, with 98.7% to 100% identity and a maximum of three single nucleotide polymorphisms (SNPs) between any two sequences. FcaGHV1 gB sequences from Ontario cats also exhibited very low genetic diversity, with 17/21 sequences being identical to the FcaGHV1 gB consensus sequence (Figure 4). There were three SNPs identified in this population: two sequences encoded a C→A change at gB nucleotide position 1690 (relative to the reference FcaGHV1 31286 strain, NC_028099.1) resulting in a H→N substitution at amino acid position 564, one sequence encoded C→G at position 1624 resulting in a L→V substitution at amino acid position 542, and one sequence encoded G→T at position 1761 resulting in a Q→H substitution at amino acid position 587 (Figure 4). These three SNPs were unique and not found in other published FcaGHV1 sequences. Collectively, these results confirm the presence of FcaGHV1 DNA in domestic cat saliva and suggest that FcaGHV1 from southwestern Ontario is genetically similar to other FcaGHV1 isolates worldwide.

### 3.3. Attempted Isolation of FcaGHV1 from Saliva in Cell Culture

**Figure 5:**
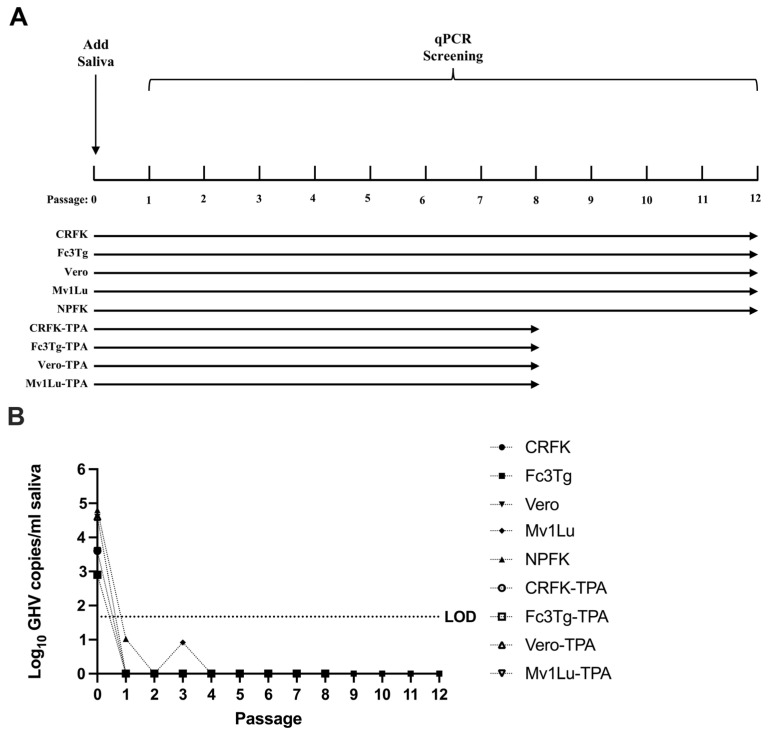
Attempted propagation of FcaGHV1 in cell culture. (**A**) Saliva positive for FcaGHV1 DNA (2.9–4.8 Log_10_ GHV copies/mL starting titer) was incubated on five cell types with weekly passaging of supernatant and cell lysate onto fresh cells. Passages on immortalized cell lines (CRFK, Vero, Mv1Lu, and Fc3Tg) were continued for 8 weeks in the presence of the phorbol ester TPA or 12 weeks without treatment. Passages on newborn primary feline kidney cells (NPFK) were continued for 12 weeks. (**B**) qPCR screening was conducted for the presence of FcaGHV1 DNA in each passage for all cell lines and treatments. However, Log_10_ GHV copies/mL saliva were indistinguishable from negative controls and below the limit of detection.

To date, infectious FcaGHV1 has not been isolated or propagated in cell culture despite several attempts utilizing infected blood cells (R.M. Troyer, unpublished data). This lack of infectious virus severely limits the potential to study FcaGHV1 infection in vitro or in vivo. In this study, we attempted to isolate infectious FcaGHV1 from saliva by a passage in cell culture using methods that have resulted in the successful propagation of related GHVs from the genus *Percavirus* [40,41,42,43]. We performed sequential passage of FcaGHV1 DNA-positive saliva in four adherent immortalized cell lines, including CRFK and Fc3Tg cells from domestic cats, Mv1Lu cells from mink that successfully propagated a closely related badger GHV, and primate Vero cells that are commonly used to isolate diverse animal viruses in a veterinary diagnostic setting [41]. We also attempted sequential passage in non-immortalized primary kidney cells from a newborn cat, referred to here as newborn primary feline kidney (NPFK) cells. For these experiments, we utilized FcaGHV1 DNA-positive saliva from the chronic shedding cat described in Figure 3 as the source material for attempted cell culture propagation (2.9–4.8 Log_10_ GHV copies/mL starting titer). We conducted blind passage of cell lysate onto fresh cells every 7 days for 8–12 sequential passages with and without the use of the phorbol ester 12-O-tetradecanoylphorbol-13-acetate (TPA) for activation of the GHV lytic cycle (Figure 5A). Based on microscopy of cell layers every 2–3 days, we did not detect viral cytopathic effect (CPE) during any of the passages. Furthermore, FcaGHV1 qPCR screening of purified cell lysate from each weekly passage was consistently below the limit of detection for this set of experiments of 46 copies/mL of supernatant/cell lysate (Figure 5B). These results suggest that if infectious FcaGHV1 is present in saliva, alternative cell lines or methods of culture will be necessary for virus propagation.

## 4. Discussion

In this study, we identified FcaGHV1 in cats for the first time in Canada and characterized the prevalence, risk factors, and sequence of FcaGHV1 DNA in feline saliva. We found that shedding of FcaGHV1 DNA in saliva is common among cats in a shelter-based population, shedding can occur consistently in a cat over two months, and feral cats had higher viral loads in saliva than non-feral cats. Viral genetic analyses indicated that FcaGHV1 gB sequences from southwestern Ontario cats were highly similar to other FcaGHV1 sequences worldwide. We also found that FcaGHV1 in saliva was resistant to viral propagation in a range of cell lines with and without phorbol ester TPA treatment.

While numerous previous studies have characterized FcaGHV1 DNA prevalence in the blood (reviewed in [25]), a recent study by Rose et al. [22] was the first to detect FcaGHV1 DNA shedding in the oral cavity. Our current study sought to build on this finding by examining, for the first time, the prevalence and quantity (viral load) of FcaGHV1 DNA in liquid saliva collected from cats. We found that 19.8% of domestic cats had detectable FcaGHV1 DNA shed in saliva, while Rose et al. found a similar prevalence of 16.8% FcaGHV1 DNA-positive oropharyngeal swabs [22]. Together, these studies suggest that cats commonly shed FcaGHV1 via the oropharynx into saliva. If FcaGHV1 is similar to other herpesviruses, such as EBV, it is likely that only a subset of infected cats actively shed the virus at any given time [44,45]. This suggests that the percentage of infected cats is likely higher than the 19.8% found to have FcaGHV1 DNA in saliva. We further found that a cat consistently shed FcaGHV1 over a two-month period, suggesting that individual cats can chronically shed FcaGHV1 in saliva over an extended period of time. These findings have potential implications for FcaGHV1 cat-to-cat transmission. With approximately one-in-five to one-in-six cats actively shedding virus at one time, this represents an abundant potential source for transmission between cats. Collectively, this suggests that FcaGHV1 transmission might commonly occur via behaviors that involve salivary contact, such as grooming and biting, though direct transmission studies are required to confirm this.

In this study, FcaGHV1 DNA load in saliva varied widely by more than 4 log_10_ copies/mL, suggesting that shedding levels may vary greatly between cats. Saliva viral load even varied in the same cat over time by up to 3 log_10_ copies/mL (Figure 3). In humans, factors associated with variability in the occurrence and level of herpesvirus salivary shedding include age, sex, immune status, and infection with other pathogens [26,46,47]. In this study, we found that cat age and sex were not associated with the occurrence or level of FcaGHV1 shedding. Intriguingly, these findings diverge from studies on FcaGHV1 prevalence in feline blood. Multiple studies have found that the presence of detectable FcaGHV1 DNA in blood is strongly associated with male sex [10,12,17,18]. The reasons for this difference are not readily apparent. FcaGHV1 PCR detection in blood and saliva likely both underestimate the true infection prevalence of the virus [19]. In the absence of a “gold standard” to determine the true infection prevalence in males and females, we can only speculate that hormonal or other sex differences may affect virus shedding in saliva or virus presence in blood. Furthermore, our study found no association between cat age and the presence or level of FcaGHV1 DNA in saliva, again in contrast to studies on blood-borne FcaGHV1 that find older age associated with the presence of FcaGHV1 DNA in blood [10,12,17,18]. However, the vast majority of cats tested in this study were young, with a median age of approximately 2 years. Studies including a larger number of older animals and/or a larger number of very young animals may have a different outcome. For instance, Rose et al. found that cats < 3 months old had a lower frequency of oropharyngeal shedding than older cats [22]. In this study, we found that feral cats had higher saliva viral load than non-feral cats. The reasons for this association are not clear, but feral cats may experience greater stress, overall poorer health, and a greater number of other infections compared to non-feral cats [48]. Previous studies have linked FcaGHV1 infection to co-infection with FIV and hemotropic mycoplasma [13,18]. Any of these factors could potentially result in greater herpesvirus reactivation from latency and subsequent shedding. However, this finding was based on a small sample size, including only 4 FcaGHV1-positive feral cats.

Sequence analysis of viral gB in this study suggests that FcaGHV1 in southwestern Ontario is genetically highly similar to FcaGHV1 found throughout the world. The high conservation of gB is not surprising, given the critical role of gB in mediating herpesvirus-cell fusion [49]. Identification of FcaGHV1 variants will likely require examination of a less conserved genomic region. The presence of several SNPs resulting in amino acid changes is intriguing, implying potential changes to gB structure and function in select viral isolates or, conversely, tolerance of these particular residues to mutation. Further studies on the structure and function of FcaGHV1 gB would be needed to elucidate any potential significance of these SNPs.

GHVs, such as those responsible for malignant catarrhal fever, can be highly resistant to cell culture propagation [50]. Likewise, FcaGHV1 has not been successfully propagated in cell culture despite several attempts utilizing infected blood cells (R. Troyer, unpublished data). Since other herpesviruses have been successfully isolated and cultured from saliva [51,52,53,54], we attempted to culture infectious viruses from cat saliva. GHVs are characterized by a relatively narrow host tropism, and like all viruses, their ability to infect and replicate is likely determined by the expression of a range of cellular factors. Since FcaGHV1′s host requirements for infection and replication have not been characterized, we chose cell lines we believed would have the best chance of being permissive to FcaGHV1 infection. We included three domestic cat cell types: CRFK, a cell line that is widely used for propagation of feline viruses [55,56]; Fc3Tg, a cat tongue cell line chosen based on its oral origin; and NPFK, primary fetal cells that presented a non-immortalized and less-differentiated cell alternative. We also included Vero cells due to their lack of functional interferon signaling and common use for the culture of diverse animal viruses [57,58]. Finally, we chose the mink lung cell line Mv1Lu due to the successful propagation of a virus closely related to FcaGHV1, mustelid GHV1 of badgers, in this cell line [41]. Our results clearly showed a lack of saliva-associated FcaGHV1 replication in these cell types—a result that was disappointing but not surprising, as there is often no clear pattern to which cells will support the propagation of a particular GHV. In some cases, the cell line that supports replication is completely unexpected, such as with the propagation of black bear GHV on human rectal tumor cells [59]. Future studies should consider employing an even broader range of target cell lines for attempted FcaGHV1 propagation. Given the lymphotropic nature of FcaGHV1 and its ability to infect both B and T cells [20], feline lymphocytes or a lymphocyte-derived cell line, such as MYA-1, may represent an alternative target cell for propagation. In consideration of GHVs’ well-documented tendency to enter into latency upon infection of the host cell, we treated our cells with the phorbol ester TPA, which is known to activate the lytic cycle of GHVs, likely through activation of protein kinase C and has been used in isolation of GHVs in previous studies [60]. In future FcaGHV1 propagation attempts, other strategies for activation of lytic replication, such as transfection of host cells with trans-activators of GHV transcription, may be useful [61]. While GHVs are considered relatively slow-growing, most can be propagated in one to several cell culture passages, so the 8–12 seven-day passages tested in this study were reasonable. However, in a few cases, GHVs have required an even greater number of passages for virus isolation [40,43], suggesting that future isolation efforts may benefit from testing additional passages. Finally, as infectious herpesviruses are very commonly shed in saliva [58,59,60,61], the presence of FcaGHV1 DNA in saliva likely indicates the presence of at least some intact virus. However, it is theoretically possible that the detected FcaGHV1 DNA could result from the shedding of infected cells or free DNA released from lysed infected cells rather than an intact virus. In future attempts, it may be possible to detect FcaGHV1 virions in saliva by electron microscopy or immunofluorescent staining for FcaGHV1 capsid proteins to confirm the presence of intact virions and further to detect virus entry using in situ hybridization to identify the viral DNA genome in target cells.

In summary, we identified FcaGHV1 in cats in Canada for the first time and found that salivary shedding of FcaGHV1 is common, with viral load in saliva being highly variable and potentially associated with feral status. These results suggest a likely key role for salivary shedding of FcaGHV1 in viral transmission that should be investigated further. While FcaGHV1 in saliva was resistant to our extensive viral propagation efforts, the establishment of a cell culture model for FcaGHV1 infection is critical to studying the biology of the virus and its pathogenic potential and should be a key target of future research efforts.

## Figures and Tables

**Table 1:** Numbers of individuals testing positive and negative for FcaGHV1 DNA in saliva for each potential predictor variable (sex, age, feral status, and health status).

Demographics	Sex		Age		Feral Status		Health Status ^1^	
	Male	Female	<1 Year	>1 Year	Feral	Non-Feral	Healthy	Non-Healthy
Positive	22	23	14	73	4	41	40	5
Negative	88	94	31	109	8	174	174	8
% Positive	20.0%	19.7%	31.1%	40.1%	33.3%	19.1%	18.7%	38.5%

^1^ Adverse health conditions are defined as upper respiratory infections, tumors, or heart conditions.

## Data Availability

The dataset is available upon reasonable request to the corresponding author.
